# King Edward's Hospital Fund for London

**Published:** 1904-05-21

**Authors:** Hugh C. Smith, John Aird

**Affiliations:** Chairman of the Executive Committee


### Editor's Letter-Box.

[Our Correspondents are reminded that prolixity is a great bar to publication, and that brevity of style and conciseness of statement greatly facilitate early insertion.]

### KING EDWARD'S HOSPITAL FUND FOR LONDON.

*To the Editor of* The Hospital.

Sir,—I enclose a letter from Sir John Aird generously offering to increase his annual subscription to this Fund by 100 guineas a year, this sum to be specially appropriated to endowment in order to raise the fixed annual income of the Fund, from investments and other sources, to £50,000 a year.

May I venture to appeal through your columns to others who may be willing to follow his example.

Yours faithfully,
Hugh C. Smith,
Chairman of the Executive Committee.

[Copy of Letter.]

14 Hyde Park Terrace, W.,
May 9th, 1904.

Dear Mr. Hugh Smith,—In reference to your letter about the King Edward's Hospital Fund for London in the *Times* of the 28th March last. Since the meeting on March 8th at Marlborough House, when his Royal Highness the Prince of Wales, President of the Fund, made the announcement that an anonymous donor was willing to give a sum, estimated to produce £4,600 a year, if others would give the further necessary sum of double that amount by the end of this year, in order to raise the annual income of the Fund from investments and other permanent sources to £50,000 a year, I have thought that the desired fund could in part be raised by annual subscriptions to be appropriated to this particular purpose, and that many would be willing to increase their annual subscriptions for so good an object.

I hope the idea may commend itself to the Executive Committee, and, if so, I shall be very pleased to become a subscriber of an additional 100 guineas.

Yours faithfully,

John Aird.

